# Sub-lethal doses of imidacloprid alter food selection in the invasive garden ant *Lasius neglectus*

**DOI:** 10.1007/s11356-022-24100-7

**Published:** 2022-11-16

**Authors:** Filippo Frizzi, Paride Balzani, Alberto Masoni, Clara Frasconi Wendt, Matilde Marconi, Asia Rossi, Giacomo Santini

**Affiliations:** 1 Department of Biology, University of Florence, Via Madonna del Piano, 6, Sesto F.No., 50019 Florence, Italy; 2 Faculty of Fisheries and Protection of Waters, South Bohemian Research Center of Aquaculture and Biodiversity of Hydrocenoses, University of South Bohemia in České Budějovice, Zátiší, 728/II, 38925 Vodňany, Czech Republic; 3 cE3c, Centre for Ecology, Evolution and Environmental Changes, Faculty of Science, University of Lisbon, Lisbon, Portugal

**Keywords:** Neonicotinoids, Ants, Invasive species, Resource selection, Binary choice, Symmetry breaking

## Abstract

Despite several restrictions to their use, neonicotinoid insecticides are still widely employed worldwide. Residual sub-lethal amounts of these chemicals can have detrimental effects on the behavior of non-target insects. Toxic effects on economically important species such as bees have been widely documented, but less is known about their toxic action on other social insects, such as ants. In this study, we assessed the effect of different sub-lethal doses of the neonicotinoid imidacloprid on the ability of colonies of the invasive ant *Lasius neglectus* to select the most profitable resource. We used Y-shaped mazes having an imidacloprid-polluted or an unpolluted sucrose solution on the two branches. Two sucrose (0.1 M, 0.5 M) and two imidacloprid (1 μg/ml, 10 μg/ml) concentrations were used. In parallel, we evaluated the marking activity of foragers who fed on the same solutions. We found that the 0.1 M sugar solution polluted with 1 μg/ml imidacloprid was significantly more frequently selected in binary choices experiments than the unpolluted resource. Moreover, the ingestion of the same combination of sugar and imidacloprid significantly increased the marking rate of foragers. The higher concentration of the pollutant had lower effects, probably because of the hormesis phenomenon. Results suggest that the lower sub-lethal dose of imidacloprid can lead ants to select again the polluted resource. This “active” selection of the pollutant may magnify the negative effects on the colonies. Due to their ecological role, any impairment of ant survival or behavior may have detrimental cascade effects on the whole ecosystem.

**Supplementary Information:**

The online version contains supplementary material available at 10.1007/s11356-022-24100-7.

## Introduction

For decades, neonicotinoids have been widely used to protect crops against insect pests. The mechanism behind the high efficiency of these neurotoxic insecticides is the tight binding of the molecule to the insect nicotinic acetylcholine receptors (nAChRs), a process that inhibits synaptic functionality and causes confusion, convulsions, paralysis, and death in the target organisms (Sheets 2010). Imidacloprid, although the widespread debate about its toxicity, is one the most employed neonicotinoids worldwide (Simon-Delso et al. 2015). Relevant residual amounts of this insecticide can be found in many agroforestry habitats, where they persist for relatively long times (e.g., Cox et al. 1997; Donnarumma et al. 2011; Bonmatin et al. 2015; Humann-Guilleminot et al. 2019; Pisa et al. 2021). These can have harmful effects on non-target organisms even at a sub-lethal concentration (Rondeau et al. 2015). Among the non-target organisms affected, honeybees have been widely studied, due to their ecological and economic relevance (Maini et al. 2010). In bees, impairment of several functions has been observed, ranging from the disruption of specific physiological functions, such as olfaction (Li et al. 2019), to the loss of orientation ability while homing (Bortolotti et al. 2003) or foraging (Yang et al. 2008). The increasing awareness of all these threats has stimulated growing concerns about the widespread use of neonicotinoids, leading the European Union to restrict their use and, from 31st of July 2022 to completely ban them (EU Regulation 485/2013; EU Commission Implementing Regulation 2017/195). However, to date, neonicotinoids are still used in approximately 120 countries worldwide (Craddock et al. 2019). In the USA, they are still extensively used (Stackpoole et al. 2021), and the Environmental Protection Agency (EPA) scheduled a Review Process for the risk assessment of several neonicotinoids, such as imidacloprid, that will end in 2024 (EPA docket ID: EPA-HQ-OPP-2008–0844).

Ants are key organisms in most terrestrial habitats and play a chief ecological role in agroecosystems (e.g., Retana and Cerdá 2000; Ottonetti et al. 2008; Brewitt et al. 2015; Diamé et al. 2018). With regard to the relationship between neonicotinoids and ant biodiversity, attention has focused on invasive ant species. Several studies assessed the effectiveness of neonicotinoids to control invasive ant species, such as the argentine ant *Linepithema humile* (Daane et al. 2008; Blight et al. 2011). In the last years, evidence is accumulating on the effects of sub-lethal doses of imidacloprid on ant behavior (Schläppi et al. 2020, 2021). For example, in the invasive fire ant *Solenopsis invicta*, the ingestion of sub-lethal concentrations (0.25 μg/ml) of neonicotinoids reduces the brood-tending ability of queens, whereas exposure to even lower amounts (0.01 μg/ml) induces increased digging activity in workers and prolonged feeding on sugary solutions (Wang et al. 2015). In an experimental maze, the foraging ability of the harvester ant *Pogonomyrmex occidentalis* was disrupted after being exposed to a low concentration of the pesticide (50 ppm), with workers struggling to find food resources, probably as a consequence of an impairment of the orientation system (Sappington 2018). Moreover, some of these behavioral effects may have important cascade consequences that extend to interspecific interactions within a community. Thiel and Köhler (2016) found that the exposure to a sub-lethal dose of imidacloprid (1 μg/ml) was sufficient to alter the competitive interactions between the dominant *Lasius niger* and the subordinate *L. flavus*, as a consequence of significant increment in the aggressiveness of the subordinate species.

Ants possess efficient collective orientation systems that allow recruiting nestmates on resources (Hölldobler and Wilson 1990). Other concurrent orientation methods can be used by some species (Wolf and Wehner 2000; Banks and Srygley 2003; Harris et al. 2007; Müller and Wehner 2010), but pheromone substrate marking is usually the key mechanism to recruit workers (David Morgan 2009). As a rule, the signal can be modulated according to the quality of the resource: the better the resource the stronger and more persistent the signal (Jackson and Châline 2007). The presence of a pheromone trail induces the recruited ants to follow it and mark the route themselves, thus reinforcing the signal. This positive feedback mechanism allows to optimize food exploitation by a colony, and when different resources are available at the same time, the best one can be exploited first. On the other hand, when two equivalent resources are available, the same mechanism generally leads to the concentration of the workforce on one of them, a phenomenon called “symmetry breaking” (Beckers et al. 1990; Sumpter and Beekman 2003). However, in some species, including the invasive ant *Lasius neglectus* (Van Loon et al. 1990), symmetry breaking does not always occurs (Frizzi et al. 2018), probably as a consequence of the huge number of workers involved in food exploitation (Cremer et al. 2008; Santarlasci et al. 2014). Given the centrality of the recruiting system in ant ecology and the key role of ants in many terrestrial ecosystems, it is important to understand how this is impaired by the presence of nicotinoids in the environment.

In this study, we used the invasive ant species *L. neglectus* as a model to assess whether exposure to sub-lethal doses of imidacloprid may interfere with resource selection. We performed field experiments based on binary choices, where ants were able to choose between two resources having the same nutritional content (sugar), but only one of them was added with a sub-lethal amount of imidacloprid, and we counted the frequency of selection of the two resources. Additionally, since the substrate marking is central in the selection of the pathway toward the resource, we recorded the marking frequency of foragers that fed on such solutions using high-resolution footage. Given the known behavioral and neurophysiological effects of sub-lethal concentrations of imidacloprid, our aim was to verify whether the neonicotinoid exposition could induce impairment in the resource selection by colonies, thus altering the natural pattern of selection between two equally nutritive resources. We might also expect that this potential alteration affected the frequency of occurrence of the symmetry breaking.

## Materials and methods

**Fig. 1:**
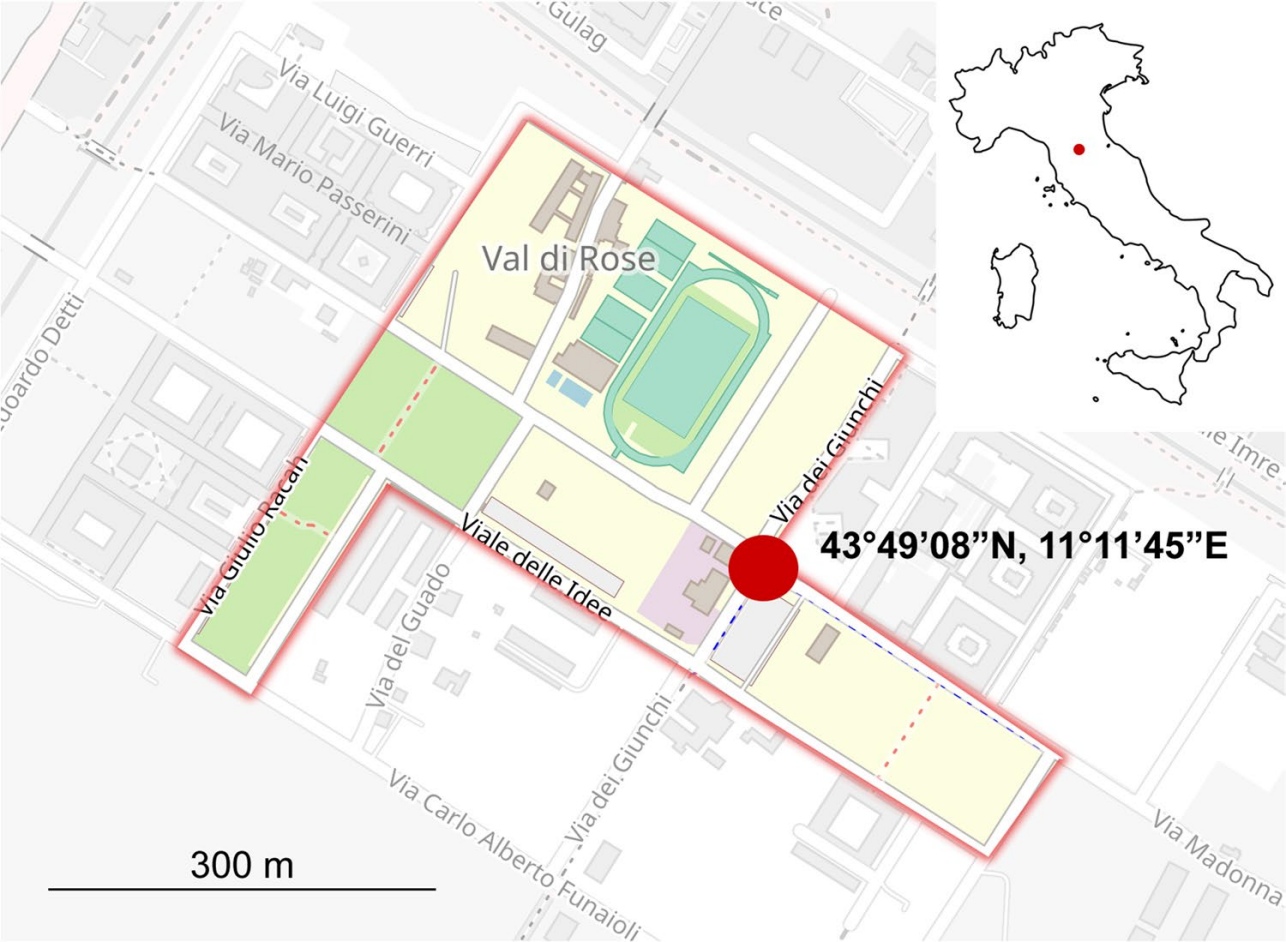
Map of the study site. The trees used in the tests were distributed aside the main streets in the red-bordered area (image elaborated from OpenStreetMap)

The study was carried out between June and July 2019 on a population of *L. neglectus* situated within the Sciences Campus of the University of Florence (43°49′08″N, 11°11′45″E, Fig. 1). The habitat is a typical suburban environment, with large buildings, roads bordered with trees and surrounded by sparse fallow fields. The climate is Mediterranean, with hot and dry summers and mild winters. The other most abundant ant species in the area is the Dolichoderinae *Tapinoma* sp. (*T. nigerrimum* group, Nylander, 1859), plus a few scattered colonies of the Myrmicinae species *Crematogaster scutellaris* (Oliver, 1972), *Messor structor* (Latreille, 1798), *Messor capitatus* (Latreille, 1798), *Pheidole pallidula* (Nylander, 1849)*,* and the Formicinae species *Camponotus aethiops* (Latreille, 1798), and *Plagiolepis pygmaea* (Latreille, 1798) (see Balzani et al. 2020). Workers of *L. neglectus* occupied the trunks of almost all the trees occurring in the area (mostly oaks, *Quercus* sp., but also cypresses, *Cupressus* spp., and pines, *Pinus* spp., Frizzi et al. 2020). Oaks scattered on an area of approximately 3.8 ha were taken as sample units.

### Preliminary tests

In the binary choice experiments, we used a concentration of imidacloprid never tested before in literature (10 μg/ml), and we performed a preliminary experiment to verify it is sub-lethal. We collected 20 groups of ants, each from a different nest hole and composed of 20 workers. Groups were housed separately in plastic containers to acclimatize to laboratory conditions without food for one night and then allowed to feed for 3 h with a sugary solution (0.5 M) polluted with 10 μg/ml of imidacloprid. The presence of this amount of imidacloprid did not significantly alter the acceptance of the solution. After 3 h, we removed the polluted source and checked mortality for a further 3 h. This time largely exceeds the time span of the binary choice experiments (see below). The same number of control tests, with ants provided with a sugary solution (0.5 M) but without imidacloprid, were also performed. Only four of the 400 ants exposed to the pollutant died, and one ant died in control tests. This difference is not significant (binomial GLM: z =  − 1.244, *P* = 0.213); hence, we considered this dose as sub-lethal for *L. neglectus*.

### Binary choice experiments

In these experiments, we offered ants two resources, which had equal sucrose content but only one of them was added with a sub-lethal concentration of imidacloprid. We used plastic Y-shaped bridges (50-cm length and 3-cm width, each branch was 30 cm long), holding a small Petri dish (3.5-cm diameter) at the end of each branch, following Frizzi et al. (2018). The angle between the two branches was 60° and optimized the ant flow on natural ant trails (Acosta et al. 1993; Jackson et al. 2004). The bridge was placed on two cylindrical smooth plastic supports coated with Fluon©, to prevent ants from climbing on the upper part. The proximal end of the bridge was bent to touch the ground at the base of a tree visited by *L. neglectus*. We used two imidacloprid (10 μg/ml and 1 μg/ml) and two sucrose (0.1 M and 0.5 M, corresponding to 3.42∙10^5^ μg/ml and 1.71∙10^6^ μg/ml, respectively) concentrations. The two sucrose concentrations were selected following previous experiments that demonstrated that *L. neglectus* is able to discriminate between 0.5 and 0.1 M sucrose concentrations, with the higher dose more frequently exploited by ants (Frizzi et al. 2018). The lower concentration of imidacloprid (1 μg/ml) was selected following Thiel and Köhler (2016) since they found it is sufficient to induce behavioral changes in two *Lasius* species.

The sucrose concentrations added with imidacloprid were used in four combinations:

- iHsH (10 μg/ml imidacloprid and 0.5 M sucrose, imidacloprid high, sucrose high).
- iHsL (10 μg/ml imidacloprid and 0.1 M sucrose, imidacloprid high, sucrose low).
- iLsH (1 μg/ml imidacloprid and 0.5 M sucrose, imidacloprid low, sucrose high).
- iLsL (1 μg/ml imidacloprid and 0.1 M sucrose, imidacloprid low, sucrose low).

**Fig. 2:**
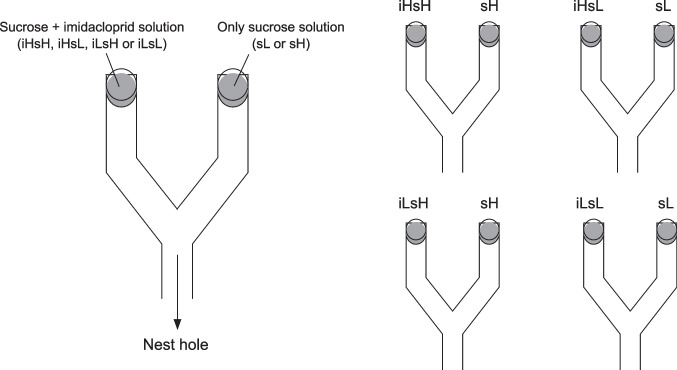
Schematic representation of the design for the binary choice experiment. On the left, is the general scheme, and on the right are the four combinations of tests. The position of the two solutions—left or right—was changed at each test. iLsL (imidacloprid low, sucrose low, 1 μg/ml imidacloprid, and 0.1 M sucrose), iLsH (imidacloprid low, sucrose high, 1 μg/ml imidacloprid, and 0.5 M sucrose), iHsL (imidacloprid high, sucrose low, 10 μg/ml imidacloprid, and 0.1 M sucrose), iHsH (imidacloprid high, sucrose high, i.e., 10 μg/ml imidacloprid and 0.5 M sucrose), sL 0.1 M sucrose, sH 0.5 M sucrose

For each combination, we performed 20 replicate tests where the solution with sucrose only (sL, 0.1 M, and sH, 0.5 M) was placed on the other branch, for a total of 80 tests. The left and right position of the polluted solution was switched each time (Fig. 2). According to Frizzi et al. (2018), in similar experiments, *L. neglectus* can select one of the resources within 60 min; thus, we chose this as experimental time. The experimental time started when the first ant reached one of the two solutions. After 60 min from the beginning, we counted the ants feeding on the two resources. To assess whether the pattern of selection was stable over time, we also recorded the same data for 120 min as a control. On the same day, we performed no more than four tests. Sampling trees were selected at least 10 m distant from one another. When a tree was selected for a test, it was not used again for at least 4 days.

### Marking frequency experiment

For this experiment, we built up a linear plastic bridge (50-cm length and 1-cm width), placed on two cylindrical supports coated with Fluon©. At one end, we placed a Petri dish (3.5-cm diameter), whereas the other end was bent to touch the ground at the base of a tree as described above. In the dish, we placed a cotton flock soaked with one of six solutions: the four combinations described above plus one low (0.1 M) and one high (0.5 M) sucrose solution as a reference. We placed a 13 MP resolution digital camera at the side of the bridge at 50-cm distance, and we filmed all ants moving on the bridge. Filming started when the first ant was observed feeding on the solution and lasted 10 min. Movies were then observed, and we counted the number of marks laid by each ant in a stretch of the bridge of 35 cm. One ant was considered to lay a mark when it flexed the gaster and touched the surface of the bridge (see Supplementary material S1). Since the frequency of marking tends to decrease with time (Beckers et al. 1993; Czaczkes et al. 2012) and the first foragers are determinant for the food selection (Frizzi et al. 2018), we subdivided the films into two stages: “early stage” (within 5 min) and “advanced stage” (after 5 min). Moreover, we recorded the direction of the movement (towards the resource or towards the nest). For each combination of the levels of these factors, i.e., stage and direction, we counted the number of marks for a minimum of 15 ants. We performed three replicates for each of the six solutions.

### Statistical analyses

In the binary choice experiments, ants could select the branch with sugar only, with sugar and imidacloprid, or both. The number of ants feeding on each dish was standardized following Frizzi et al. (2018): for each test, a dish was scored as “selected” if the number of ants exceeded 60% of the total number of ants in the two dishes. The alternative dish was scored “avoided.” When none of the dishes attracted more than 60% of ants, we scored the outcome of the test as “no selection.” Hence, the frequencies of the three choices summed to one for each of the four test solutions (iHsH, iHsL, iLsH, iLsL). To analyze differences between frequencies, we used binomial generalized linear models (GLM), with the test solution as the main factor. Tukey post hoc tests between pairwise different resources were used for multiple comparisons. As a control, the same analysis was also performed with data at 120 min.

To assess the effect of the stage (early or advanced), direction, and type of solution on the marking frequency of foragers, we fitted Poisson generalized linear mixed models (GLMMs), with the direction of movement (Dir, toward nest or toward food), the type of resource (Res, the different test solutions), and the stage (Stage, early or advanced) as fixed factors and video id as a random term. The effect of these terms was assessed by ranking 14 different models of increasing complexity by their AIC and then testing the significance of the terms included in the best model.

Subsequently, we compared the number of marks of ants that fed on the polluted resources and on the reference sugary solutions, focusing on the differences between the solutions used in the binary choice experiments. Differences were assessed by Poisson GLMM models and Tukey post hoc tests between the resources of interest. Models included the type of resource as a fixed term and the replicate id as a random term. Analyses were carried out with the R 4.0.3 software (R Core Team 2020), using the “lme4” package (Bates et al. 2015). In all tests, the significance threshold was *α* = 0.05.

## Results

### Binary choice experiments

**Fig. 3:**
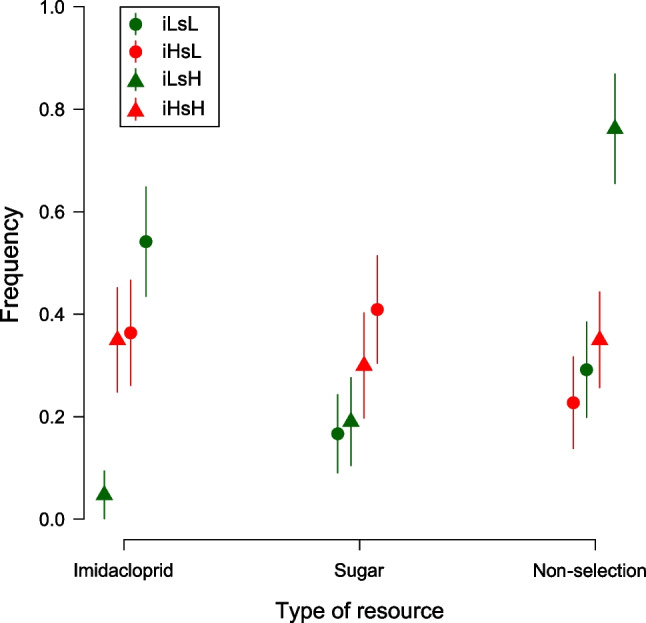
GLMM predictions of the frequency of selection of one of the two sides (imidacloprid and sugar) and of non-selection in binary choice experiments. Error bars are the standard errors. iLsL (imidacloprid low, sucrose low, 1 μg/ml imidacloprid, and 0.1 M sucrose), iLsH (imidacloprid low, sucrose high, 1 μg/ml imidacloprid, and 0.5 M sucrose), iHsL (imidacloprid high, sucrose low, 10 μg/ml imidacloprid, and 0.1 M sucrose), iHsH (imidacloprid high, sucrose high, i.e., 10 μg/ml imidacloprid and 0.5 M sucrose)

The models revealed an effect of the type of solution, whereas for the frequency of selection of the unpolluted side the type of resources did not have any detectable effect (Table 1 and Fig. 3). Post hoc tests revealed significant differences between iLsL and iLsH in the frequency of selection of the imidacloprid side. Moreover, the frequency of no selection was different between iLsL and iLsH, and between iLsH and iHsL, with a borderline effect between iLsH and iHsH (Table 1). As for the probability of choosing the imidacloprid side, with the higher imidacloprid concentration, the polluted side was chosen in about 40% of cases. On the contrary, when imidacloprid concentration was low, the polluted side was chosen approximately 60% of the times when sugar concentration was also low, but was clearly not selected (chosen in less than 20% of cases) when sugar concentration was high (Fig. 3). The frequency of selection of the unpolluted side was slightly lower for lightly polluted resources, but this difference was not significant (Fig. 3). Finally, the frequency of non-selection was always nearly 35%, except when imidacloprid concentration was low and sugar high (iLsH), where the frequency of non-selection was about 80% (Fig. 3). Equivalent results have been recorded after 120 min from the beginning of the experiment (Supplementary material S1).

**Table 1:** Results of complete models for binary choice experiments and multiple comparisons between types of resource in pairs. In bold significant tests (α ≦ 0.5)

Imidacloprid side			
**Global test: χ**^**2**^** = 14.87, df = 3, p = 0.002**			
Comparison	z value	p	
iHsL—iHsH	0.09	1.00	
iLsH—iHsH	− 2.11	0.14	
iLsL—iHsH	1.26	0.57	
iLsH—iHsL	− 2.18	0.12	
iLsL—iHsL	1.20	0.61	
**iLsL—iLsH**	**2.87**	**0.02**	
Sugar side			
Global test: χ^2^ = 4.22, df = 3, p = 0.239			
Non-selection			
**Global test: χ**^**2**^** = 15.76, df = 3, p = 0.001**			
Comparison	z value	p	
iHsL—iHsH	− 0.87	0.82	
**iLsH—iHsH**	**2.57**	**0.05**	
iLsL—iHsH	− 0.41	0.98	
**iLsH—iHsL**	**3.31**	**0.01**	
iLsL—iHsL	0.50	0.96	
**iLsL—iLsH**	** − 3.01**	**0.01**

### Marking frequency experiment

**Fig. 4:**
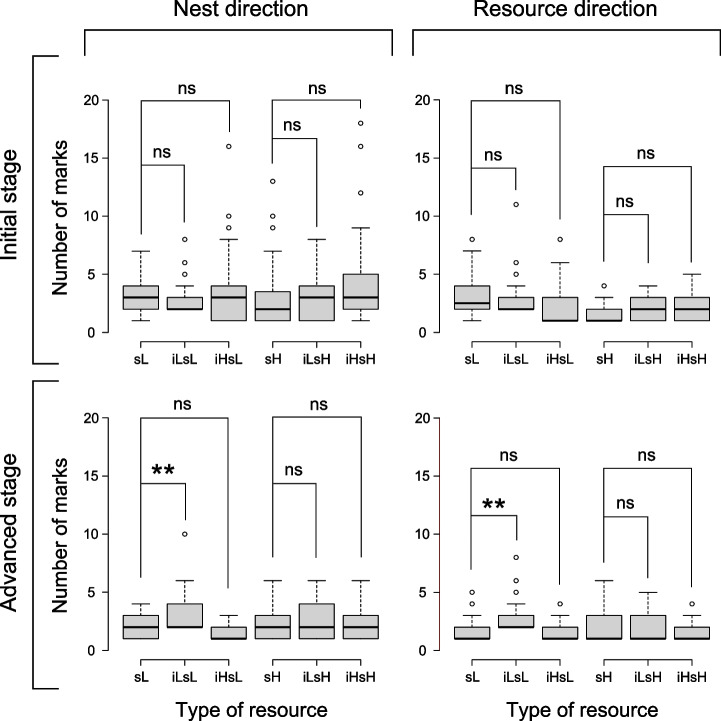
Number of marks laid by ants feeding on the different experimental solutions, subdivided by direction of movement and stage. Whiskers are the maximum values, except for outliers (circles). Bold line is the median. Type of resource: iHsH (imidacloprid high, sucrose high, i.e., 10 μg/ml imidacloprid and 0.5 M sucrose), iHsL (imidacloprid high, sucrose low, 10 μg/ml imidacloprid, and 0.1 M sucrose), iLsH (imidacloprid low, sucrose high, 1 μg/ml imidacloprid, and 0.5 M sucrose), iLsL (imidacloprid low, sucrose low, 1 μg/ml imidacloprid, and 0.1 M sucrose) and two sugary solutions without imidacloprid, sL (sucrose low, 0.1 M sucrose) and sH (sucrose high, 0.5 M sucrose). The lines connecting the boxes represent Tukey’s HSD pairwise tests (significance threshold *α* = 0.05). Significance level: ns, not significant, **p < 0.01

We counted 1365 marking ants in total. The model that best fitted the complete marking dataset is the one including the direction of movement, the stage, and their interaction (Table 2). The complete model, which also included the type of resource, had a similar AIC (ΔAIC = 0.290). Given the interaction between stage and direction, both of which significantly affected the number of marks, we tested the differences among the types of resources for all four combinations of stage and movement direction. The number of marks laid for each of these combinations is shown in Fig. 4. During the initial stage, the presence of imidacloprid did not alter the marking frequency with respect to the solution with sucrose only, neither toward the nest nor toward food. On the contrary, in the advanced stage, a significantly higher number of marks was recorded only between the solution with the low concentration of sugar and the one including the low concentration of imidacloprid, both for nest direction (Wald *χ*^2^ = 6.98, *d.f.* = 1, *p* = 0.008) and resource direction (Wald *χ*^2^ = 8.13, *d.f.* = 1, *p* = 0.004).

**Table 2:** AIC values of models used for both the binary choice and marking experiments. In bold the lowest AIC

Marking frequency experiment		
Model	AIC	ΔAIC
Null	4733.54	79.18
Dir	4667.13	12.77
Stage	4726.52	72.16
Res	4737.92	83.56
Res + Dir	4671.64	17.28
Res*Dir	4661.08	6.72
Dir + Stage	4660.27	5.91
Dir*Stage	**4654.36**	-
Res + Stage	4728.89	74.53
Res*Stage	4732.38	78.03
Dir + Res + Stage	4662.83	8.48
Dir + Res*Stage	4666.92	12.56
Dir*Stage + Res	4656.79	2.43
Dir*Stage*Res	4654.65	0.29

Imid, selection of the imidacloprid-added resource; Sucr, selection of the solution with sucrose only; No Sel, no selection. Null, null model; Res, type of resource; Dir, direction of movement (nestward or foodward). The asterisk means that the model includes both factors and their interaction. In bold the model with the lowest AIC

## Discussion

The results of our study show that the ingestion of sub-lethal doses of the neonicotinoid imidacloprid may alter the resource selection ability of *L. neglectus*, leading to a higher selection of a polluted resource and the increase of the marking frequency by foragers who fed on it. Both the sugar and pollutant concentrations appeared to play a role in selection since only the solution with low content of both imidacloprid and sucrose concentrations (1 μg/ml and 0.1 M respectively) was selected with higher frequency (approximately 50–60% of tests). This solution was also the one that induced significantly more frequent pheromone marks in the trail-marking experiments, although only in the advanced stage. This finding contrasts with the known decrease of the marking activity in time when the substrate is already rich in pheromone (Czaczkes et al. 2014). The mechanism behind this behavior is still to be clarified but, potentially, two possible explanations could be proposed. First, this effect might be due to an exciting action of imidacloprid on ants, inducing workers to increase the marking rate and consequently to select the polluted part more. Although variations of the behavior of ants exposed to imidacloprid have been already recorded, for example, an increased aggressiveness of subordinates (Thiel and Köhler 2016), no current evidence or previous findings support this hypothesis. A second possibility is that imidacloprid affects the ant ability to detect the pheromone on the substrate, not reducing therefore the marking frequency when the substrate is saturated by chemical traces (Beckers et al. 1993; Czaczkes et al. 2014). Olfactory impairment due to imidacloprid ingestion is not a novelty in hymenopterans, as it has been observed in *Apis mellifera* (Yang et al. 2012; Williamson and Wright 2013). Whatever the mechanism, the action of the pollutant appeared to be very rapid since the altered marking frequency is present even in ants that had just fed on polluted resources. This quick effect has been also previously found by Thiel and Köler (2018). Moreover, also the foragers moving from the nest showed an altered marking frequency, suggesting that these ants had been in contact with the pollutant before leaving the nest. The rapid diffusion of pollutants through trophallaxis has been demonstrated several times in ants and is the basis of the chemical control of ant pests through baiting (e.g., Soeprono and Rust 2004; Camargo et al. 2017; Buczkowski 2019; Buczkowski and Wossler 2019).

The ingestion of the solution with the same low imidacloprid concentration but higher sugar content did not cause any significant alteration of the marking rate of the ants. However, in the binary choice experiment, this combination led the colony to collectively select one of the two resources only a few times (lower than 20% of the total), exploiting both resources. In *L. neglectus*, the “symmetry breaking” (i.e., the collective selection of only one of two identical resources) occurs in approximately 80% of trials when both resources are equal (Frizzi et al. 2018), a proportion roughly respected in trials made with the three other tested solutions (iLsL, iHsL, iHsH). An interesting feature of the resource selection in ants is that when the nutritional values of two alternative resources are high for both (as for example the solutions with a high concentration of sugar in these experiments), the proportional difference between the chemical signals deposed by ants in the two pathways is smaller than if the two resources were scarce; thus, the symmetry breaking occurs less frequently (Price et al. 2016). In our experiments, a possible alteration of the olfactory system might impair the foragers’ ability to distinguish between two similar traces when both resources had a high concentration of sugar. Hence, foragers could select at random one of the two branches, and this would disrupt the feedback mechanism at the base of the symmetry breaking. A future physiological investigation on the interaction between imidacloprid and the olfactory system in ants may help to address this point.

Finally, the fact that the solutions containing higher doses of imidacloprid did not have any particular effect on both the binary selection and the marking frequency might seem surprising. In principle, we would have expected higher concentrations to cause stronger behavioral alteration than lower amounts of imidacloprid. This apparent incongruence might depend on opposite physiological responses according to the dose of the toxicant, a process called hormesis, which is not uncommon in insects (Cutler 2013; Guedes and Cutler 2014; Cutler et al. 2022). When an organism is subjected to a stressor that disrupts its homeostasis, it reacts with defensive mechanisms to re-establish optimal conditions. Hormesis can be seen as an overcompensation response of the organism to low stresses triggered only when a toxicant exceeds a threshold dose (Calabrese 2001; Guedes and Cutler 2014). This process is transversal across many taxa and may depend on numerous different physiological mechanisms (Calabrese 2013; Rix and Cutler 2022). It is possible that the higher concentration of imidacloprid entailed overcompensation defensive systems, therefore mitigating the behavioral effects. However, to our knowledge, this is the first evidence of this process in ants, suggesting that more accurate investigations need to be conducted.

In conclusion, with this study, we demonstrate for the first time that sub-lethal amounts of a neonicotinoid can alter the collective resource selection ability in ants, determining a greater frequency of selection of a polluted resource with respect to an unpolluted one. This “active” selection bias toward polluted sources can amplify the effects of contamination. Interestingly, it appears that lower imidacloprid concentrations are more effective in altering the foraging behavior than higher—though sub-lethal—concentrations, raising some questions about the physiological effects of this pesticide on ants. While here we tested the effects of neonicotinoids on the foraging behavior of an invasive ant species, similar results may be found for other native ant species with a similar ecology and foraging behavior to *L. neglectus*, suggesting likely harmful effects on native ant communities. This last finding increases the concern about the dangerousness of the residuals of this chemical in the environment for the ants, because a radical behavioral change in the species may have unpredictable ecological cascade effects.

## Supplementary Information

Below is the link to the electronic supplementary material.

Supplementary file1 (DOCX 33 KB)

## Data Availability

Data and materials are available on request.
