# Supplementary material for: Sub-lethal doses of imidacloprid alter food selection in the invasive garden ant Lasius neglectus
Source: Environ Sci Pollut Res Int. 2022 Nov 16;30(10):27501–9. doi: 10.1007/s11356-022-24100-7 (PMC9995417; doi:10.1007/s11356-022-24100-7)
Supplement: Supplementary file 1 — Supplementary file1 (DOCX 33 KB) [file 11356_2022_24100_MOESM1_ESM.docx]

**Supplementary Information SI1**: Model predictions of the frequency of selection of one of the two sides (imidacloprid and sugar) and of non-selection in the binary choice experiments, 120 min from the beginning of the tests. In the table multiple comparisons among different type of resources are reported. In the figure, error bars are the standard errors. iLsL (imidacloprid Low, sucrose Low, 1 μg/ml imidacloprid and 0.1 M sucrose), iLsH (imidacloprid Low, sucrose High, 1 μg/ml imidacloprid and 0.5 M sucrose), iHsL (imidacloprid High, sucrose Low, 10 μg/ml imidacloprid and 0.1 M sucrose), iHsH (imidacloprid High, sucrose High, i.e. 10 μg/ml imidacloprid and 0.5 M sucrose).


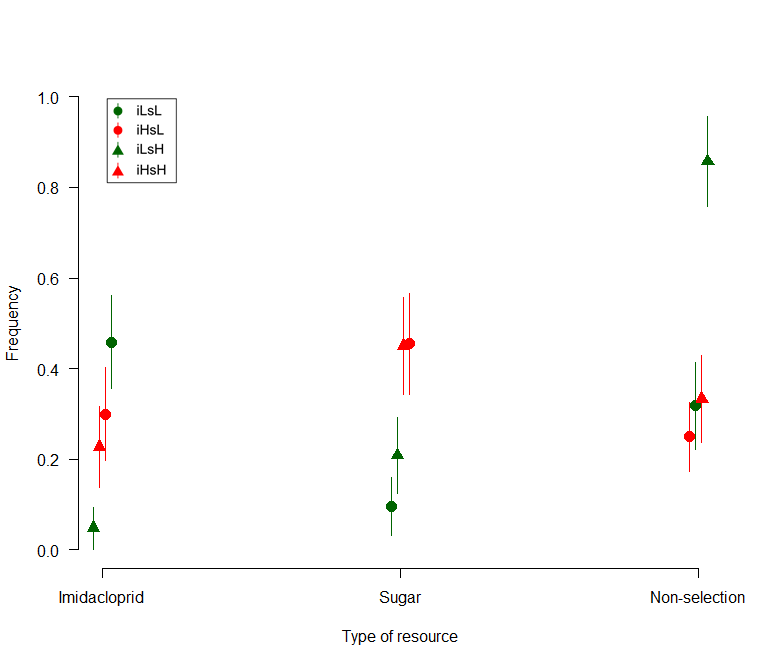


| Imidacloprid selection | | |  |  |  |  |
| --- | --- | --- | --- | --- | --- | --- |
|  |  |  |  | z value | *P* value |  |
| iHsL | - | iHsH | == | -0.534 | 0.9486 |  |
| iLsH | - | iHsH | == | -1.893 | 0.2211 |  |
| iLsL | - | iHsH | == | 1.068 | 0.6995 |  |
| iLsH | - | iHsL | == | -1.549 | 0.3958 |  |
| iLsL | - | iHsL | == | 1.618 | 0.3564 |  |
| iLsL | - | iLsH | == | 2.563 | **0.0474** | * |
|  |  |  |  |  |  |  |
|  |  |  |  |  |  |  |
| Sugar selection | |  |  |  |  |  |
|  |  |  |  | z value | *P* value |  |
| iHsL | - | iHsH | == | 0.03 | 1 |  |
| iLsH | - | iHsH | == | -2.361 | 0.0828 |  |
| iLsL | - | iHsH | == | -1.682 | 0.3279 |  |
| iLsH | - | iHsL | == | -2.412 | 0.0728 |  |
| iLsL | - | iHsL | == | -1.746 | 0.295 |  |
| iLsL | - | iLsH | == | 1.021 | 0.7331 |  |
|  |  |  |  |  |  |  |
|  |  |  |  |  |  |  |
| Non-selection | |  |  |  |  |  |
|  |  |  |  | z value | *P* value |  |
| iHsL | - | iHsH | == | 0.488 | 0.96155 |  |
| iLsH | - | iHsH | == | 3.57 | **0.00182** | ** |
| iLsL | - | iHsH | == | 0.602 | 0.93099 |  |
| iLsH | - | iHsL | == | 3.301 | **0.00511** | ** |
| iLsL | - | iHsL | == | 0.109 | 0.99953 |  |
| iLsL | - | iLsH | == | -3.273 | **0.00583** | ** |
